# Management of Persistent Left Superior Vena Cava: Overcoming Lead and Vascular Complications With Transcatheter Pacing System Implantation

**DOI:** 10.7759/cureus.67754

**Published:** 2024-08-25

**Authors:** Maria A Rodriguez-Santiago, Hector E Sepulveda, Juan C Sotomonte

**Affiliations:** 1 Cardiology, University of Puerto Rico, Medical Sciences Campus, San Juan, PRI; 2 Medicine, University of Puerto Rico, Medical Sciences Campus, San Juan, PRI; 3 Electrophysiology, Cardiovascular Hospital of Puerto Rico, San Juan, PRI

**Keywords:** lead fracture, persistent left superior vena cava, vascular anomaly, leadless pacemaker, transcatheter pacing system

## Abstract

Isolated persistent left superior vena cava (PLSVC) is a rare congenital anomaly typically found incidentally due to its asymptomatic nature. However, it can present technical challenges for device implanters. We report a case involving a patient with PLSVC, for whom the implantation of a transcatheter pacing system proved to be the most effective long-term solution. Although this venous anomaly initially provided a safe pacing route, it eventually led to early complications. The patient, a 78-year-old Puerto Rican man with hypertension, diabetes mellitus, and complete atrioventricular block, experienced multiple complications with pacing devices. After a failed left-sided pacemaker implant, a right-sided single-chamber ventricular device was placed, but it led to right ventricular lead fractures and was eventually abandoned. A new pacing system implanted in the left chest lasted only a year. Venography revealed a patent PLSVC with a previously implanted device now obstructed by an occluded left brachiocephalic vein. After laser-assisted extraction, a dual-chamber device was successfully implanted through the PLSVC. Despite unremarkable physical and lab results, the patient later showed syncope and high lead impedances with fractures in both leads and total PLSVC occlusion. A transcatheter pacing system was chosen to address the complex anatomical issues and abandoned hardware. Atrial synchronized pacing was confirmed the morning after implantation, and the patient was safely discharged. Ensuring a stable ventricular rhythm is crucial for patients with complete heart block. When hemodynamic stability is compromised by recurrent lead fractures and rare anatomical variants, implanters must consider alternative solutions. In this case, a transcatheter system was selected to avoid further lead and pocket-related complications and mitigate the risks of additional laser-assisted extractions. At the end of the device’s lifespan, a new device can be implanted without significant anatomical issues, and the epicardial route remains a viable option if necessary.

## Introduction

Isolated persistent left superior vena cava (PLSVC) is a rare congenital anomaly where the vein originates at the junction of the left subclavian and internal jugular veins, traverses the left side of the mediastinum, and drains into the right atrium (RA) via the coronary sinus (CS) [1]. The most recent classification system is categorized into three types: type I represents normal venous anatomy; type II represents the presence of a PLSVC in the absence of a right superior vena cava (R-SVC); and type III indicates the presence of both R-SVC and PLSVC. Subsequently, it can be divided into type 3A which is dual-SVC connected to the brachiocephalic vein, and type 3B dual-SVC not connected to the brachiocephalic vein [2]. PLSVC forms when the primitive venous system, particularly the left common cardinal vein (CCV) and the caudal part of the left anterior cardinal vein, fails to regress during embryological development, resulting in the persistence of the PLSVC [3]. Typically, the PLSVC drains into the RA through the CS. Nonetheless, it can directly drain into the left atrium via the unroofed CS in 20% of cases [3]. PLSVC occurs in 0.3-0.5% of the population and poses technical challenges for cardiac device implantation [4].

During the embryological stage, particularly around the fifth week of gestational age, three sets of veins play a crucial role: the cardinal veins, vitelline veins, and umbilical veins. Some of these veins will regress and become remnants, while others persist and form important vascular structures [1]. Normally, a single SVC is present on the right side of the mediastinum. This SVC is formed by the fusion of the right CCV and the caudal part of the right anterior cardinal vein. Both veins drain into the sinus venosus, which eventually directs blood to the primitive atrium [3]. The left vitelline vein regresses completely, while the right vitelline vein persists and contributes to the formation of part of the inferior vena cava (IVC) [1].

Normal embryological development leads to the formation of a typical venous system: a single SVC on the right side, which receives poorly oxygenated blood from both the right and left brachiocephalic veins. Each brachiocephalic vein is formed by the union of the internal jugular vein and the SCV.

We present a case of a patient with a PLSVC, where a transcatheter pacing system proved to be a lifesaving solution. Previous device placements through this venous anomaly offered a safe pacing route for several years but also predisposed the patient to early system failures and complications.

This article was previously presented as a poster at the 2023 ACC American College of Cardiology Annual Scientific Meeting on March 6, 2023.

## Case presentation

**Figure 1:**
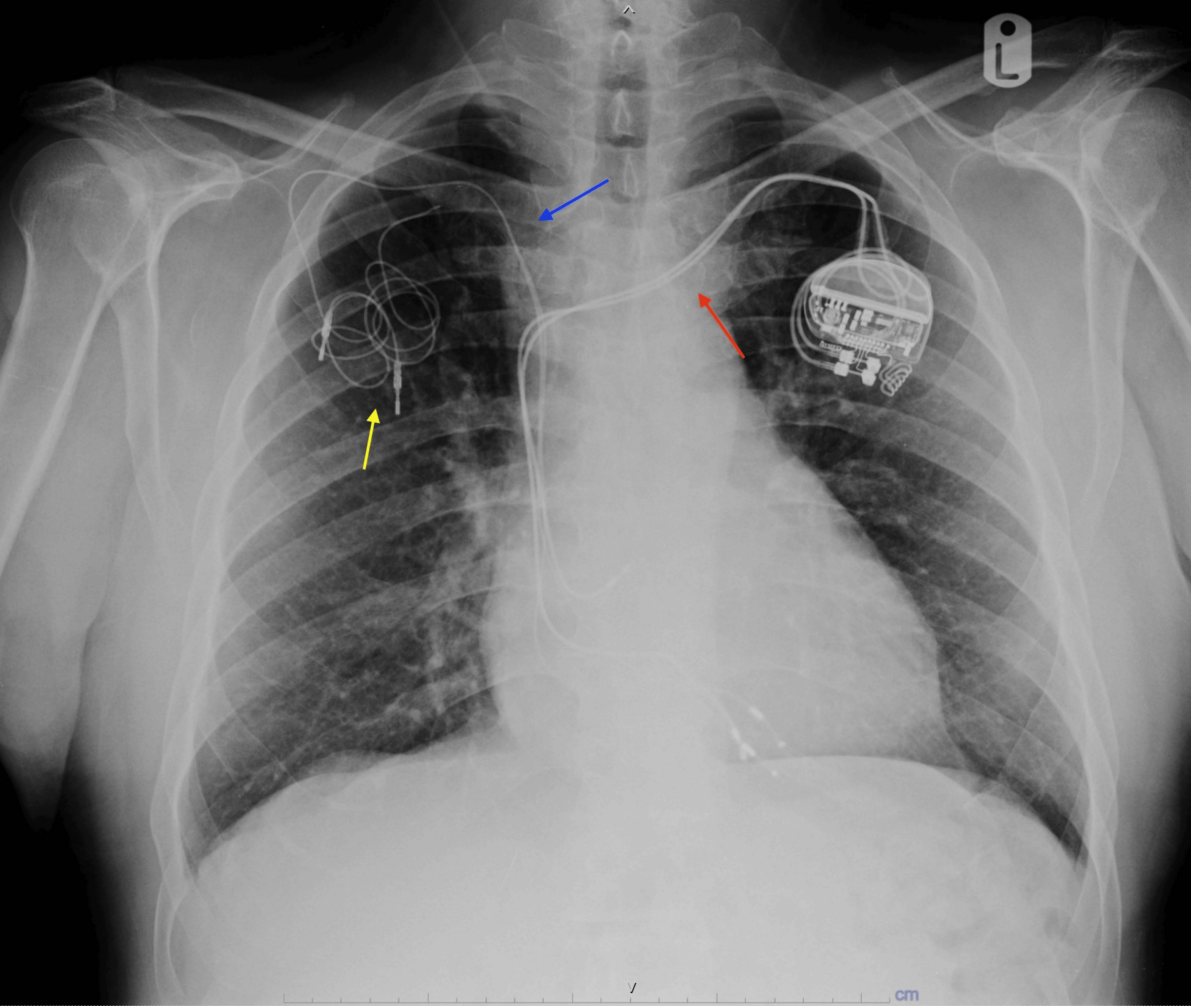
Chest X-ray showing abandoned right-sided leads and newly implanted left-sided pacing leads. The right ventricle failed lead extraction is indicated by the coiled lead (yellow arrow). Abandoned right ventricular leads are shown (blue arrows). The new dual-chamber device features right atrial and right ventricular leads passing through the left brachiocephalic vein (red arrows).

This case involves a 78-year-old Puerto Rican man with hypertension, diabetes mellitus, and a complete heart block first diagnosed after presenting to our institution with a syncope. An initial attempt to implant a left-sided pacemaker failed, leading to the implantation of a right-sided single-chamber ventricular device. Six years later, the right ventricular lead fractured, and after a failed extraction attempt, it was abandoned, and a new lead was implanted on the same side (Figure 1).

**Figure 2:**
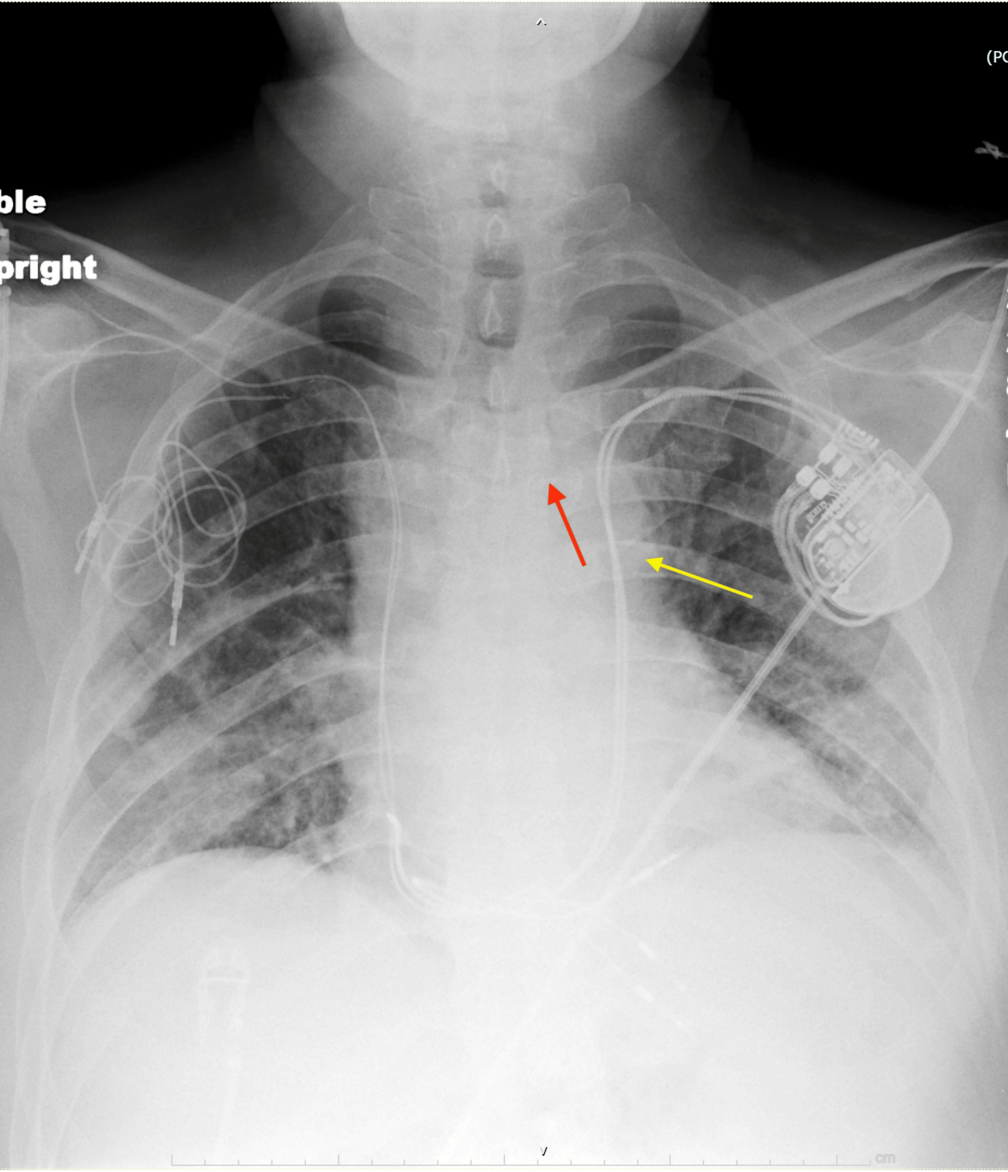
Chest X-ray showing the newly implanted left-sided lead passing through the PLSVC. The left-sided dual chamber device with right atrial and ventricular leads passing through the left brachiocephalic vein was removed (red arrow). A new dual chamber device with right atrial and ventricular leads was implanted through the PLSVC (yellow arrow). PLSVC: persistent left superior vena cava

Nine years later, the functioning right-sided lead failed, necessitating the implantation of a new pacing system in the left chest through the left brachiocephalic vein, which malfunctioned shortly after one year. Venography during the subsequent revision at our center revealed a PLSV, with the in-situ device implanted via the now totally occluded left brachiocephalic vein. After the laser-assisted extraction of the left-sided system, a new dual-chamber device was implanted through the PLSVC (Figure 2).

**Figure 3:**
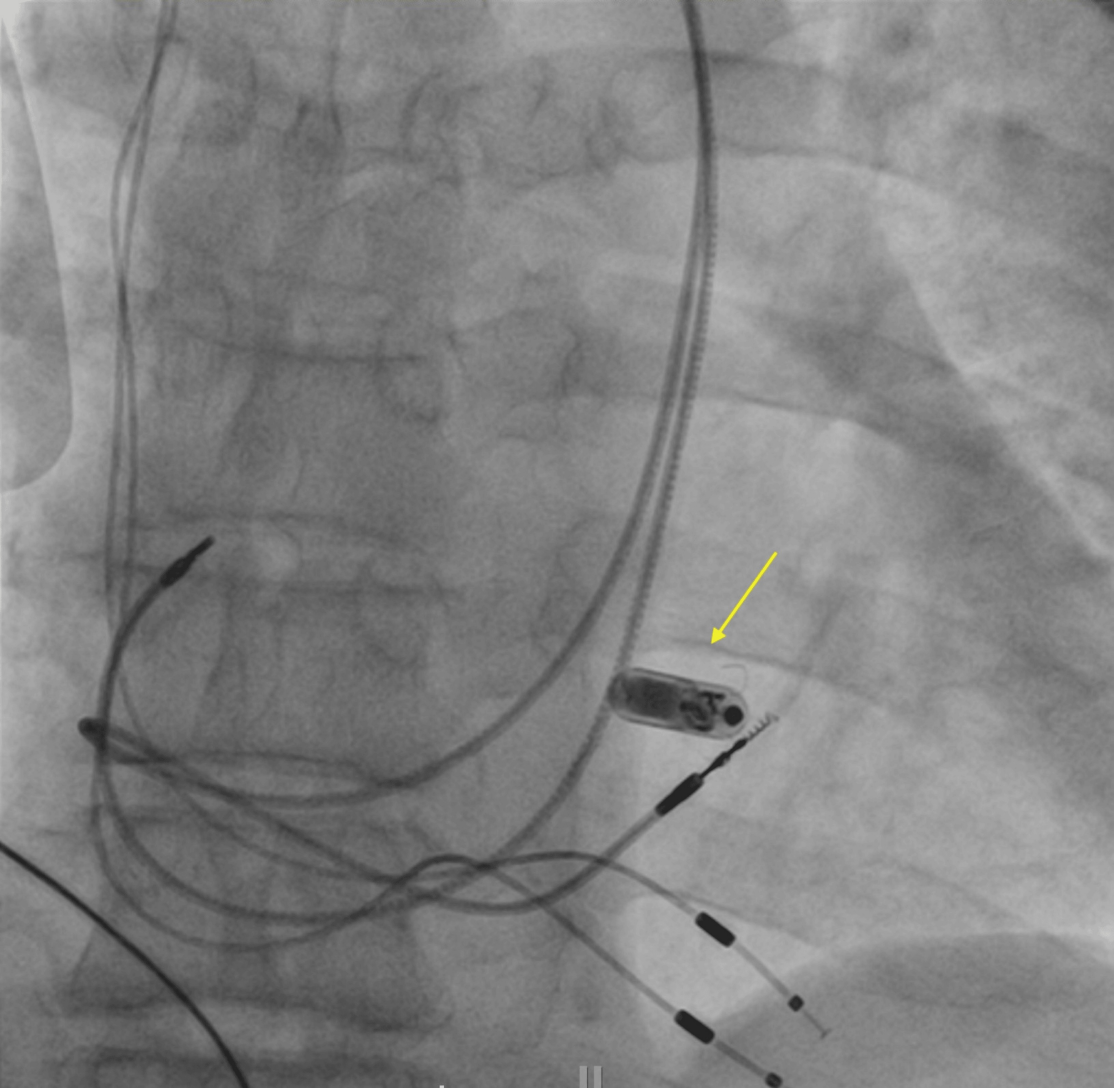
Fluoroscopic image showing the deployment of a transcatheter leadless pacing system (yellow arrow).

Three years later, the patient presented with syncope and high lead impedances. An X-ray examination revealed fractures in both leads and total occlusion of the PLSVC. Considering the patient’s anatomy and the presence of multiple abandoned hardware, a transcatheter pacing system was implanted (Figure 3).

**Figure 4:**
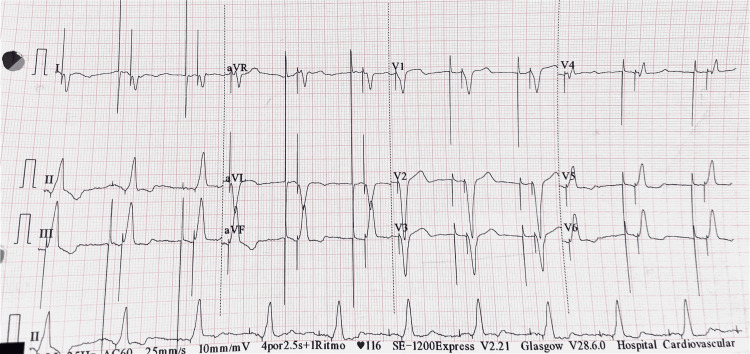
Electrocardiogram with atrial and ventricular paced morphology in DDD mode. DDD: dual chamber pacing, sensing, and response to stimuli

Atrial synchronized pacing was confirmed after implantation, and the patient was safely discharged home (Figure 4).

## Discussion

PLSVC has been linked to cardiac arrhythmias due to abnormal drainage into the CS, which can cause atrial enlargement and compression of the conduction system, including the atrioventricular (AV) node and the bundle of His [1]. When anatomical variants like PLSVC and recurrent lead fractures pose challenges to traditional transvenous pacing systems, the strategic use of transcatheter pacing systems offers a reliable solution for providing ventricular pacing in patients with complete AV block. Typically, a permanent device, such as a pacemaker or defibrillator, is implanted on the patient's nondominant side, usually, the left side, using the left subclavian and left brachiocephalic veins to access the right side of the heart. However, some literature suggests that in patients with PLSVC, cannulation of the left subclavian vein to reach the right side of the heart should be avoided, as these patients may experience higher-than-normal right atrial pressures and anatomical irregularities that can lead to mechanical complications [3].

Contralateral lead failures, such as those observed in this case, may be linked to reduced dimensions of the R-SVC. Additionally, some cases indicate that the primary challenge of using the PLSVC is the risk of dissection or perforation of the thin-walled CS [5]. Conversely, other complications can include unsuccessful attempts to advance the lead from the CS through the tricuspid valve into the right ventricle [6]. According to Razi et al., it is easier for the implanter to cross the tricuspid valve in a patient with a PLSVC type II compared to a PLSVC type III because the catheter only needs to navigate a single major pathway rather than managing multiple venous entrances, such as RSVC-RA, which confers a higher anatomical complexity [7].

Most of the time, PLSVC is found incidentally in view of their asymptomatic nature. PLSVC can be diagnosed by bilateral venography, chest CT scan, or echocardiography with agitated saline [8]. Being aware of the anatomy is important because preoperative evaluation may reduce the risk of complications, timing of the procedure, and radiation exposure. Adkis et al. addressed the most common complications encountered during cannulation of the CS through a PLSVC, which include lead dislodgment, and the need for intra-operative bilateral procedures [8].

We chose to implant a transcatheter system to minimize lead- and pocket-related complications and reduce the risk of repeated laser extractions. At the end of the device's life, a second device can be implanted without significant anatomical compromise, and the epicardial route remains a viable option if needed. Ensuring a stable ventricular rhythm is the primary goal of pacing therapy for patients with complete heart block. When recurrent lead fractures are compounded by rare anatomical variants, hemodynamic stability can be jeopardized, prompting the need for alternative solutions.

## Conclusions

PLSVC is an uncommon and usually asymptomatic vascular anomaly that can lead to cardiac arrhythmias. While device placements through this type of venous anomaly may offer a safe pacing route for several years, they also increase the risk of early system failures and complications. Transcatheter leadless pacing systems become a crucial lifesaving option when other alternatives with similar risk-benefit profiles have been exhausted.
